# Cryo-TEM simulations of amorphous radiation-sensitive samples using multislice wave propagation

**DOI:** 10.1107/S2052252521008538

**Published:** 2021-09-30

**Authors:** Benjamin Himes, Nikolaus Grigorieff

**Affiliations:** a RNA Therapeutics Institute, University of Massachusetts Chan Medical School, 368 Plantation Street, Worcester, MA 01605, USA

**Keywords:** cryo-TEM, multislice wave propagation, *cis*TEM, frozen plasmon method

## Abstract

Image simulation plays a central role in the development and practice of high-resolution electron microscopy, including transmission electron microscopy of frozen-hydrated specimens (cryo-EM). Simulating images with contrast that matches the contrast observed in experimental images remains challenging, especially for amorphous samples. Current state-of-the-art simulators apply *post hoc* scaling to approximate empirical solvent contrast, attenuated image intensity due to specimen thickness and amplitude contrast. This practice fails for images that require spatially variable scaling, *e.g.* simulations of a crowded or cellular environment. Modeling both the signal and the noise accurately is necessary to simulate images of biological specimens with contrast that is correct on an absolute scale. The ‘frozen plasmon’ method is introduced to explicitly model spatially variable inelastic scattering processes in cryo-EM specimens. This approach produces amplitude contrast that depends on the atomic composition of the specimen, reproduces the total inelastic mean free path as observed experimentally and allows for the incorporation of radiation damage in the simulation. These improvements are quantified using the matched filter concept to compare simulation and experiment. The frozen plasmon method, in combination with a new mathematical formulation for accurately sampling the tabulated atomic scattering potentials onto a Cartesian grid, is implemented in the open-source software package *cis*TEM.

## Abstract 2

This work describes cryo-TEM simulations of amorphous radiation-sensitive samples using multislice wave propagation, and by introducing the ‘frozen plasmon’ method. Combined with a coarse-grained all-atom solvent model, this accurately models inelastic scattering effects leading to radiation damage, solvent motion and contrast loss.

## 1. Introduction   

The quantitative understanding of image contrast is an important goal in cryo-EM to enable accurate measurement of sample densities, optimize image processing strategies for high-resolution reconstruction of macromolecular structures and refine models of image formation. The experiments presented in this paper address a simple question: is it possible to simulate images of biological macromolecules embedded in amorphous ice with the correct contrast? To answer this question, we examine several definitions of contrast that share the common goal of describing how well the ‘signal’ stands out from the ‘noise’, defining signal-to-noise ratios. The key to using these metrics is defining how the variances of the signal and noise are affected by different sources of error, including numerical errors, specimen motion during imaging, radiation damage and the dependence of amplitude contrast on atomic species. We begin by defining sources of noise.

The power (variance) of the noise in cryo-EM images outweighs the power of the signal, often by a factor of 20 or more. The dominant source of noise in cryo-EM is ‘shot’ noise, arising from the stochastic nature of detecting an electron at a given location and time due to low-dose imaging conditions. A detailed analysis by Baxter *et al.* (2009 ▸) demonstrated the need to also consider structural noise, defined as any contrast arising from sources other than the final object of interest: carbon film, crystalline ice, radiation-damaged particles, unwanted macromolecular conformers, the supporting amorphous ice, *etc*. Unlike shot noise, structural noise is affected by objective lens aberrations, which give rise to the contrast transfer function (CTF). Baxter *et al.* modeled both the structural noise and the shot noise as additive white Gaussian noise, which fails to capture the artifacts and challenges commonly encountered during image processing, as previously demonstrated by Scheres *et al.* (2007 ▸).

An improvement in how the structural noise is simulated, particularly that arising from the supporting amorphous ice, can be found in *TEM Simulator* (Rullgård *et al.*, 2011 ▸) and *InSilicoTEM* (Vulović *et al.*, 2013 ▸). They implement multislice wave propagation as described originally by Cowley & Moodie (1957 ▸), resulting in noise that is affected by the CTF. The result of a multislice simulation is a probability distribution defined by the squared complex modulus of the electron wavefunction at the detector ψ_detector_(*x*,*y*). The simulated image is then formed by drawing from a Poisson distribution unique to every pixel while incorporating the influence of the detector quantum efficiency (DQE).

Most of the information transferred from the specimen to the image in high-resolution cryo-EM is captured in phase contrast arising from interference between the unscattered wave and the wave representing electrons elastically scattered by the specimen; ignoring higher-order interactions between scattered waves is known as linear image formation theory. A secondary form of contrast, amplitude contrast, is present due to electrons lost because they scatter outside the objective lens aperture or loss of electrons from the elastic image due to inelastic scattering. The latter source of amplitude contrast is enhanced using an energy filter (Yonekura *et al.*, 2006 ▸). Unlike phase contrast, amplitude contrast cannot be explained by linear image formation theory (Erickson, 1973 ▸) and is accounted for *post hoc* via a phase shift term added to the CTF applied to the simulated image (Erickson & Klug, 1971 ▸). This treatment is also common practice in solving the inverse problem of image reconstruction, which seeks to answer the question ‘what is the probability of the model given the observed data’. However, in forward modeling, which asks ‘what is the probability of observing some data given a particular model’, it is desirable to account for the fact that amplitude losses depend on both atom type and local mass thickness. For example, elastic scattering outside the objective lens aperture is more probable for heavy atoms, like gold, than light atoms like carbon. This heavy/light atom trend is inverted for amplitude contrast arising from inelastic losses, as the ratio of inelastic:elastic scattering probability is higher for light atoms (Egerton, 1976 ▸; Reimer & Ross-Messemer, 1989 ▸) such that they produce greater amplitude losses in energy-filtered images than heavy atoms. To understand how this affects a simulated image, we first discuss how amplitude contrast is currently accounted for in multislice simulations.

The multislice formalism is essential for thick specimens where the projection approximation fails, as it incorporates important effects like multiple scattering of electrons and the curvature of the Ewald Sphere. Increasingly thick samples are also less transparent to electrons, and all simulators we are aware of apply an implicit ‘energy filter’ to remove inelastically scattered electrons from the final image. To account for inelastic losses, a single thickness parameter is used to attenuate the image intensity according to

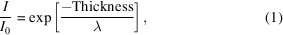

where *I*_0_ is the unattenuated image intensity and λ is the inelastic mean free path for single scattering – the average distance an electron passes through the specimen before being scattered inelastically at least once. It is clear that these single filters cannot work for specimens with variable mass thickness (*e.g.* at the edge of a cell) or for variable atomic composition (*e.g.* the increased phospho­rus concentration in the nucleus). Even purified single-particle samples with a limited subset of atomic species have two very different environments that need to be simulated: the molecule and the solvent. We will refer to how well the molecule stands out from the solvent as the ‘solvent signal-to-noise-ratio’ SNR_solvent_ as quantified by Yonekura *et al.* (2006 ▸) where *I* is the mean image intensity and σ_solvent_ is the standard deviation in the solvent region:

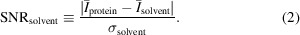

Typically, the solvent is modeled by a single value given by the mean inner potential for aqueous water and added on top of the simulated molecules in projection. This approach, which we will refer to as ‘the continuum model’, is equivalent to using an infinite time average of a collection of moving water atoms. One shortcoming of the continuum model is the failure to account for the hydration radius of a molecule, which should be zero inside a particle, higher than the bulk solvent immediately outside the particle envelope and gradually falling off with distance (Shang & Sigworth, 2012 ▸). Ignoring the fact that molecules displace the solvent has been shown to produce SNR_solvent_ that fails even visual inspection at exposures of 100 e^−^ Å^−2^ (Vulović *et al.*, 2013 ▸).

We now know that the infinite time average used in the continuum model does not adequately describe reality; even though the solvent is frozen low-density amorphous ice (LDA), it is not static during the imaging process. McMullan and Henderson quantified the motion of water molecules in LDA during imaging, estimating an RMSD of ∼1 Å/e^−^ Å^−2^ (McMullan *et al.*, 2015 ▸). Importantly, this motion results in a blurring of the solvent contrast over time, which can be thought of as low pass filtering, and so σ_solvent_ decreases with increasing exposure. The net result is that SNR_solvent_ is a function of the total exposure in an image, gradually increasing as the solvent becomes more blurred. Of note, the increase of SNR_solvent_ with exposure is further amplified in experimental images by mass loss, which also decreases σ_solvent_ and increases the numerator in Equation (2) by reducing 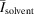 . A more sophisticated version of our solvent model may implement this mass loss in future work.

While SNR_solvent_ is useful for its simplicity, a more detailed analysis requires another metric to quantify how well simulated images recapitulate experimental images. For this, we propose using the matched filter, which is the statistically optimal realization of a cross-correlation detector. With image statistics characteristic of cryo-EM data, the output of the matched filter can be simply defined as the ratio of the cross-correlation coefficient (*CCC*) to the standard deviation of the *CCC* when only noise is present (σ_*n*_) (Rickgauer *et al.*, 2017 ▸) including any sources of structural noise as defined above.

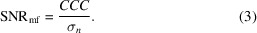

The upper bound on the SNR_mf_ is given by the ratio of the power of the input signal to the power of the noise in the image (McDonough, 1995 ▸). This means, for example, that a larger molecule will generally have a higher SNR_mf_, while any disagreement between the signal in the image and the simulated template reduces the SNR_mf_ from this maximal value. As such, the relative accuracy to which the simulated molecular density matches experimental data can be determined by searching images using a matched filter. To evaluate Equation (3), we use the cross-correlation tools and relevant preprocessing available in *cis*TEM (Grant *et al.*, 2018 ▸; Lucas *et al.*, 2021 ▸).

The shortcomings of the continuum model stem from a disregard for the changes in the sample during imaging. Both radiation damage to the molecule of interest and sample motion are the result of energy being transferred to the specimen via inelastic scattering. For frozen amorphous samples, inelastic scattering is generally attributed to plasmons, *i.e.* collective excitation of valence electrons by the electric field of the imaging electrons. However, the extent to which these are bulk plasmons, which are strongly delocalized, or more localized single-electron excitations remains unclear (Egerton, 2011 ▸). Independent of the exact form of the plasmons, their net effect is an alteration of the system’s Hamiltonian during imaging, such that the product of a traditional multislice simulation, ψ_detector_(*x*,*y*), is no longer valid. Just as the original multislice method introduced a division of the specimen potential into thin spatial slices to ensure the small-angle approximation is valid, we suggest dividing the simulated exposure into small temporal slices, where the specimen does not change too much. While we refer to time here, what is most practical from the point of view of the microscopist is exposure measured in e^−^ Å^−2^. Therefore, the time step in our simulator is specified as the desired exposure per movie frame. Exposure-rate-dependent phenomena like detector DQE and beam coherence are parameterized by an exposure rate with the exposure time implicitly set by the software according to the user-supplied exposure per frame divided by the exposure rate.

## 2. Theory   

There are three main components in modeling the image formation process in high-resolution transmission electron microscopy (HRTEM) of which cryo-EM is a subset: (1) the relativistic electron wavefunction and its modulation by the sample; (2) the exposure-dependent Coulomb potential of the specimen; (3) the microscope, including apertures, detector, lens optics and aberrations.

In this work, we are concerned primarily with how the Coulomb potential changes due to energy being deposited in the specimen during imaging and will provide only a summary of the other two components. The interested reader is referred to, in increasing order of completeness, the treatments by Vulović *et al.* (2013 ▸), Kirkland (2006 ▸), Reimer & Kohl (2003 ▸) and Hawkes & Kasper (2018 ▸).

Unlike photons, electrons have a spin quantum number and so their interaction with matter is governed by solutions to the Dirac equation. Given reasonable approximations (Hawkes & Kasper, 2018 ▸), a relativistically corrected version of the Schrödinger wave equation, called the Klein–Gordon equation, is used in practice. Analytical solutions to this equation are intractable for all but the simplest systems, so we turn to multislice wave propagation (Ishizuka & Uyeda, 1977 ▸), which produces an approximate numerical solution to this equation. The first step in a multislice simulation is the calculation of the specimen’s projected Coulomb potential 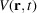 ; the time dependence will be subsequently omitted assuming a quasi-stationary solution for exposure to a single electron. The potential is divided into thin slices along the imaging axis, which can be approximated by two-dimensional scattering potentials through which the electron wavefunction is sequentially propagated. This subdivision ensures the potential varies slowly in the direction of the electron wave propagation, such that the small-angle approximation remains valid and scattered spherical wavefronts may be approximated locally by a parabola (Fresnel diffraction). In the limit of infinitely thin slices, this results in an *exact* numerical solution to the Klein–Gordon equation (Goodman & Moodie, 1974 ▸).

Multislice simulations can model both elastic and inelastic scattering processes, provided that the respective Coulomb potentials can be calculated. In analogy to the optical potential in light microscopy, inelastic scattering is incorporated into the wave theory via a complex term in the specimen potential 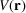 as introduced by Slater (1937 ▸).

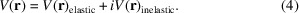

The isolated atom superposition approximation states that the specimen potential *V*(**r**) may be represented as the sum of the individual atomic potentials φ(**r**)_*i*_. We introduce a scaling factor β to compensate for the contribution of bonds among those atoms to maintain the correct total scattering cross section:

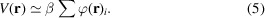

The elastic atomic potential can be calculated using relativistic Hartree–Fock wavefunctions (Doyle & Turner, 1968 ▸). The solutions for isolated atoms, having isotropic distributions, are commonly parameterized by a sum of four or five Gaussian functions (Peng *et al.*, 1996 ▸). Typically, the potential is recorded indirectly as these fits are tabulated as elastic electron scattering factors 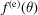 , defined as the Fourier transform of the elastic potential (Peng, 1999 ▸):

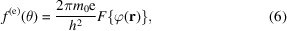

where θ is the scattering angle, e is the electron charge, *m*_0_ is the rest mass, *h* is the Planck constant and *F* denotes the Fourier transform operator. An important relation we will return to later relates the spectral distribution of the scattering factor to the differential scattering cross section – the probability density of an electron being scattered through a solid angle Ω: 

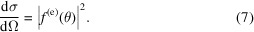

Though it is straightforward to calculate from first principles, 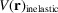 is more problematic given the varied mechanisms with which an incident electron may transfer energy to the specimen: ionization, excitation, dissociative attachment, vibrational and rotational excitations, bremsstrahlung, *etc*. (Plante & Cucinotta, 2009 ▸). An example where 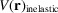 is well defined is for radiation-insensitive crystalline specimens, where thermal diffuse scattering (TDS) caused by phonon excitation is the primary contributor to the complex potential (Peng *et al.*, 1996 ▸). One model used to calculate the TDS potential treats the time-averaged atomic displacement through Debye–Waller factors and improves the accuracy of dynamic RHEED calculations (Dudarev *et al.*, 1995 ▸). This time-averaged approach is analogous to how the solvent calculation, specimen motion, radiation damage and alignment errors are accounted for in HRTEM simulations of biological specimens by *B*-factors, which are related to Debye–Waller factors by a factor of 4.

While this time-averaged approach preserves the total intensity of the projected interaction potential (Kirkland, 2016 ▸), it is well known that the image contrast produced in this way is systematically wrong, often by a factor of three or more. The error, known as the Stobbs factor (Hÿtch & Stobbs, 1994 ▸), becomes worse with increasing strength of the electron specimen interaction which, in turn, depends on the average mass thickness in the specimen. Stobbs *et al.* proposed two likely causes for the observed contrast mismatch between simulation and observation: (a) existing simulators do not fully account for radiation damage to the specimen and/or (b) they fail to model the inelastic scattering with sufficient accuracy. As recently shown empirically, these are related phenomena (Peet *et al.*, 2019 ▸).

Van Dyck *et al.* demonstrated that the Stobbs factor could be largely corrected by the ‘frozen phonon’ method (Van Dyck, 2009 ▸, 2015 ▸). The approach is conceptually simple: a series of simulations are carried out where each atom is displaced randomly, drawing from a probability distribution based on empirical TDS values. The *intensities* in the image plane as calculated from these individual simulations are then averaged together. Here we propose a similar idea, applied to radiation-sensitive frozen-hydrated specimens, where plasmons are the primary form of inelastic scattering. The frozen plasmon method presents several computational and theoretical challenges: (1) the number of solvent atoms, O(10^9^), greatly outweighs those of the macromolecules we wish to simulate images for, O(10^5^), requiring careful algorithmic design to make the computations tractable. (2) The solvent and macromolecules have very different elastic and inelastic total scattering cross-sections, as well as different average mass densities [∼0.94 g cm^−3^ for low-density amorphous ice and ∼1.38 g cm^−3^ on average for proteins (Fischer *et al.*, 2004 ▸)], meaning that the amplitude contrast and inelastic losses cannot be applied *ad hoc* to the final simulated image and must be considered on a per-atom basis. (3) The preceding points also place a requirement on the accuracy of the calculation of each atomic scattering potential, which can no longer simply be rescaled and so must be correct from the start.

The scattering factor for plasmons in low-density amorphous ice is needed to achieve the appropriate contrast, which depends on the appropriate spectral distribution. To obtain an expression 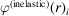 , we start from the double differential scattering cross-section for plasmons (Ferrell, 1956 ▸; Egerton, 2009 ▸). The essential form is Lorentzian

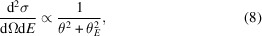

with the angular dependence θ and the energy dependence captured in the characteristic angle,

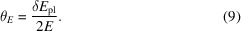




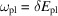
 is the energy lost to the plasmon and *E* the kinetic energy of the incoming electron. To calculate a scattering factor for plasmons, we first form an empirical probability distribution for plasmons arising from singly scattered electrons in amorphous ice, derived from EELS data published by Du & Jacobsen (2018 ▸). We then numerically integrate Equation (8) over energies in the low-loss spectrum (7.5–100 eV) for each angle. The low-energy cutoff was chosen to coincide with typical energy slit widths for Gatan energy filters (15–20 eV), which fails to exclude ∼3% of plasmons, whereas the higher energy cutoff excludes ∼1% of plasmons.

We then combine this spectrum with empirical measurements of the ratio of inelastic to elastic total scattering cross sections, which are inversely proportional to the atomic molecular weight (Reimer & Ross-Messemer, 1989 ▸). As we calculate the elastic potential during simulation, we separately accumulate an inelastic potential scaled per atom by these total probabilities. During wavefunction propagation, this inelastic potential is given the correct Lorentzian form, taken to be the square root of the values above.

**Figure 1:**
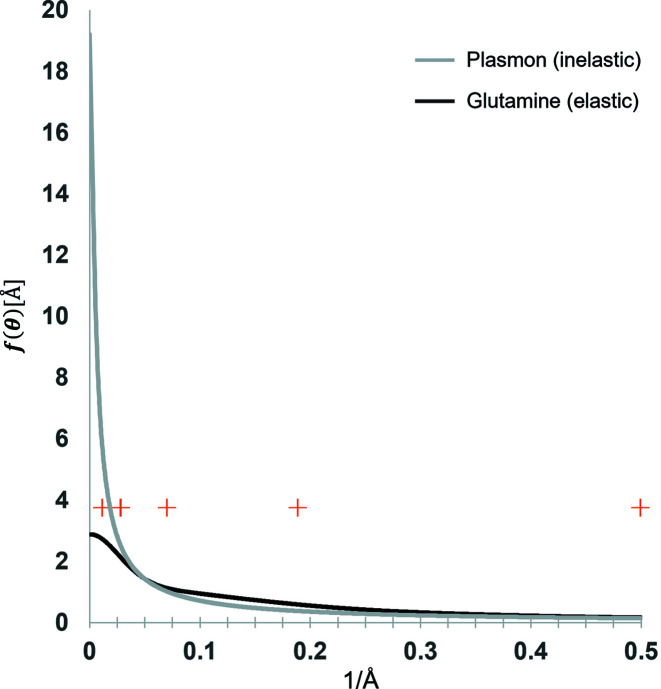
Elastic scattering factor for a glutamine molecule (black line) versus the inelastic scattering factor for a plasmon pseudo-particle (grey line). Orange markers indicate the right edge of bins compromising 20% of the band-limited scattering probability for the inelastic plasmon scattering factor as calculated from the squared norm of the scattering factor.

Plasmons scatter strongly at low angles and are generally referred to as being delocalized. This is reflected in Fig. 1 ▸ where the inelastic scattering factor we derived for plasmons is compared with the elastic scattering factor for a glutamine molecule. While plasmon scattering dominates at low resolution compared with elastic scattering, it still contributes significantly at high angles as can be seen by the orange hash marks in Fig. 1 ▸ that demarcate bins of 20% total inelastic scattering probability out to the Nyquist frequency, 2 Å^−1^ in this example. The precise nature of inelastic scattering in amorphous materials is not well understood, such that the relationship between this high-resolution information and the underlying specimen structure is not defined.

## 3. Results   

### 3.1. Accurate representation of molecular density   

For isolated neutral atoms, the scattering potential, defined as the Fourier transform of the parameterized scattering factors, can be written as

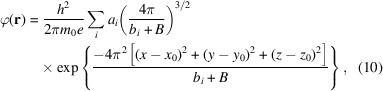

where *a_i_* and *b_i_* are the fit coefficients, *B* is the Debye–Waller factor, and the other symbols have the same meaning as elsewhere. This atomic potential is sharply peaked about the atom coordinates ( 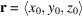 ) in real space, requiring a high sampling rate when discretizing to maintain the total projected potential. This high sampling rate effectively produces a numerical integration of Equation (10). To allow for coarser sampling, and hence improve the computational efficiency of our simulator, we analytically integrate the expression from Equation (10):

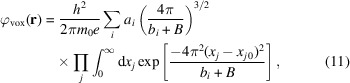

resulting in

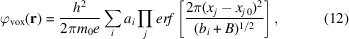

Here *erf* is the standard error function, and the vox subscript indicates the value is over a discrete voxel, and *x_j_* indicates the *x*, *y* and *z* coordinates. Though the potential in each voxel is marginally more costly to calculate (to evaluate the limits of integration, the error function must be evaluated six times per voxel, compared with a single exponential), this is more than compensated by the reduced number of voxels needed. For example, simulating at 0.5 Å voxel pitch is 125× less computationally expensive than simulating at 0.1 Å voxel pitch. Though the voxel pitch is the same in the *z*-dimension, the slab thickness is a free parameter which also affects computational efficiency. A simple test to determine the maximum allowable thickness, as suggested by Kirkland (2006 ▸), is to search for the point where the results of the simulation become dependent on slab thickness. Our simulations begin to show a dependence on slab thickness around 7 Å (data not shown) and, therefore, we typically use 5 Å. Even more important than computational speed, using Equation (12) in our simulations also means the sampled potential still has the correct magnitude and is not simply proportional to the continuous potential, as discussed in the following section.

### 3.2. Compensating for the isolated atom superposition approximation   

While the integral formulation of the scattering potential in Equation (12) preserves the calculated potential of all the individual atomic contributions, there is still a systematic underestimation of the scattering potential due to bonding interactions. This is generally estimated to be between 5–10% of the total potential (Peng *et al.*, 2010 ▸), and ignoring this difference is referred to as the isolated atom superposition approximation. Given that we want to obtain images that are quantitative on an absolute scale, we sought to measure and calibrate this error. To approximate the redistribution of the scattering potential due to bonding in a biological specimen, we use the available data for amorphous carbon, comparing with results from electron holography as follows. The average phase shift in a material depends on the mean inner potential of the material (*V*_0_), the thickness (*t*) and an interaction constant *C*_E_ (Reimer & Kohl, 2003 ▸),

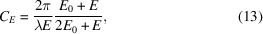

where *E*_0_ and *E* are the rest energy and kinetic energy of the imaging electron with wavelength λ. Additionally, surface boundary effects are also known to be important in cryo-EM imaging, so we compared our calculated phase shift δφ with empirical results obtained using electron holography, which measures both the mean inner potential of carbon (*V*_0_ = 9.04 eV for 1.75 g cm^−3^ density) and an additional thickness-independent surface-induced phase shift φ_add_ (0.497 radians) (Wanner *et al.*, 2006 ▸):

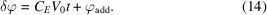

**Figure 2:**
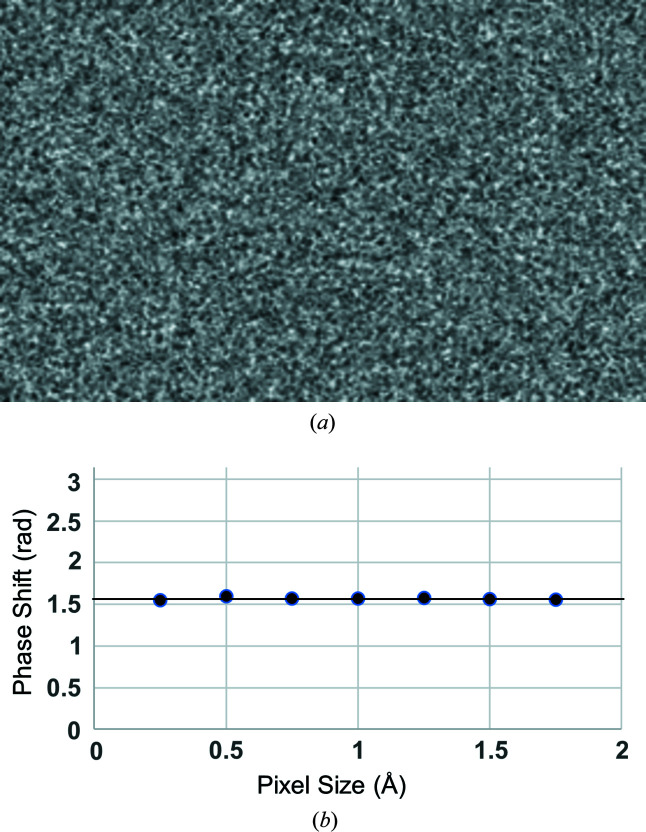
(*a*) Phase plate simulated from an amorphous layer of carbon atoms 348.6 Å thick with a density of 1.75 g cm^−3^. (*b*) Mean phase shift for the simulated phase plate as a function of pixel sampling rate during simulation. Black line plotted at π/2 radians for visual reference.

Considering the principle of a Zernike phase plate, we simulated an amorphous carbon sheet that should produce a phase shift of π/2 radians [Fig. 2 ▸(*a*)] with a density of 1.75 g cm^−3^ and 348.6 Å thickness per Equation (14). Our simulation suggested that the average phase shift is ∼3.8% too small. To correct this error, we introduce a constant scaling factor [Equation (5)] of 1.038 to the isolated atomic potentials. The simulated phase plate also serves as a sanity check to show that the calculation of the elastic scattering potential is consistent across different pixel sizes [Fig. 2 ▸(*b*)].

### 3.3. Modeling the bulk solvent   

Simulating the bulk solvent is computationally demanding due to the sheer number of water molecules in a biological sample. We elected to calculate a coarse-grained model for water, where each water molecule is represented as a single isotropic scattering center. We based the elastic scattering factor for our pseudo-waters on the elastic scattering factor tabulated for oxygen but scaled by the ratio of the total elastic scattering cross-section of oxygen:water, which we know from experiment (Plante & Cucinotta, 2009 ▸). These pseudo-molecules are seeded randomly at the proper density for low-density amorphous ice (∼0.94 g cm^−3^). A movie is then simulated, where each time step (movie frame) is defined by a user-specified exposure, and the specimen is held constant within that time.

Amplitude losses due to inelastic scattering are incorporated into the multislice formalism via a complex scattering potential, commonly defined as linearly proportional to the real (elastic) potential, for example as in *InSilicoTEM*. A detailed analysis of why this proportional model is inadequate is found by Dudarev and coworkers (Peng *et al.*, 1996 ▸). Rather than using a linearly proportional model, we derive the complex scattering potential from the bulk solvent elastic potential rescaled to have a power spectral density (PSD) based on our plasmon scattering factor as defined in the theory section.

**Figure 3:**
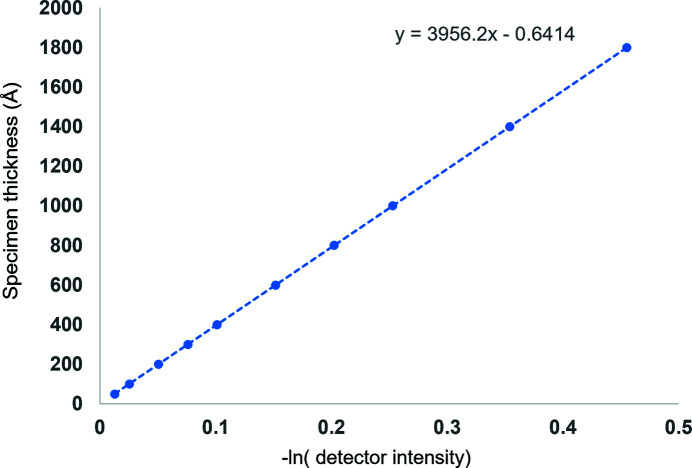
Coarse-grained all-atom solvent model in combination with the inelastic scattering factor for plasmons we derived produces amplitude losses via the complex potential that do not need to be scaled *post hoc*. The slope is a readout for the inelastic (single-scatter) mean free path in our simulated solvent.

Referring to Fig. 3 ▸, we observe the expected functional form for the attenuation due to inelastic scattering.

### 3.4. Amplitude contrast   

**Figure 4:**
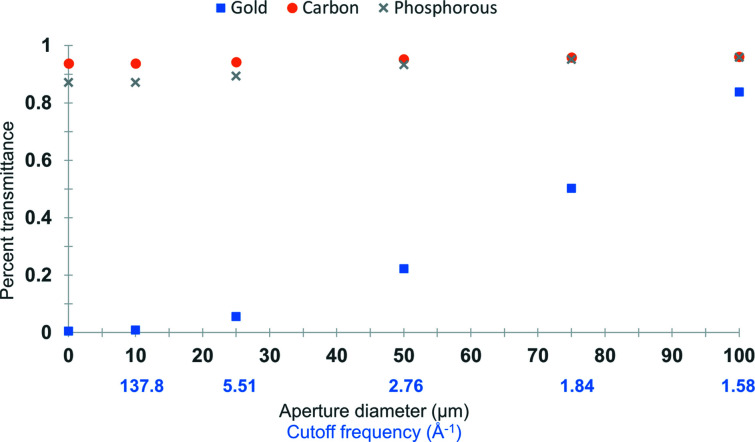
Scattering outside the objective lens aperture generates amplitude loss that varies based on atomic species. The smallest value calculated here (0.01 µm) is a pr­oxy for scattered versus not scattered electrons. A simulated amorphous carbon layer (as in Fig. 1 ▸) scatters a total of ∼6.2% incident electrons elastically. Replacing the carbon atoms with gold atoms, ∼99.5% of electrons are scattered.

Before leaving the discussion of bulk properties of amorphous samples, we now examine the other form of amplitude contrast arising from electrons being scattered outside the objective lens aperture. This is incorporated in the simulation by applying an aperture function directly to the complex wavefunction prior to image formation, which results in an attenuation of the expected number of electrons at the detector. This is demonstrated in Fig. 4 ▸ for a series of aperture diameters and a simulated amorphous specimen with density and thickness as used previously for the ‘phase plate’, with atomic potentials for carbon (orange circles), phospho­rous (gray x’s) or gold atoms (blue squares). The smallest aperture used (0.01 µm) excludes all but the unscattered beam, and so is a measure of the total transmittance of the simulated layer.

### 3.5. Accurate representation of solvent noise   

In the preceding sections, we assessed the behavior of large collections of a single type of atom. A more rigorous demonstration that the contrast being simulated is correct on an absolute scale is to compare groups of atoms with different scattering properties: the solvent and the solute. To do so, we use Equation (2) as a metric to quantify the SNR_solvent_ calculated by both the continuum model and the frozen plasmon method, as compared with experiment.

**Figure 5:**
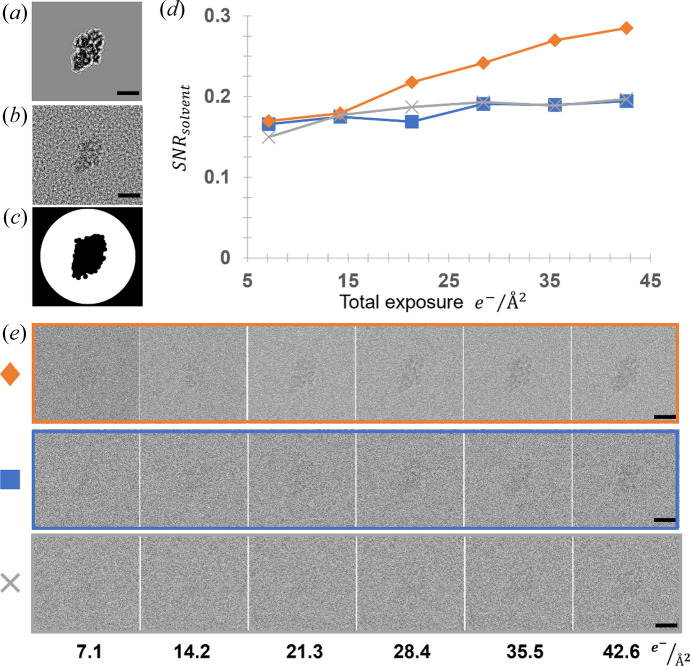
Comparison of the continuum and coarse-grained all-atom solvent model with the experimental data. (*a*) ∣ψ_detector_∣^2^ with a constant potential added for the solvent. (*b*) ∣ψ_detector_∣^2^ with coarse-grained all-atom water model. The average intensity in (*a*) and (*b*) in the solvent region is identical within numerical precision. (*c*) Mask used in calculating SNR_solvent_ [Equation (2) main text] where the white region was used for the solvent, and the central black region for the protein. (*d*) Plot of SNR_solvent_ as a function of accumulated exposure for experimental data (blue line, square marker), coarse-grained all-atom solvent model (gray, x marker) and constant solvent potential (orange diamond marker). (*e*) Images used in calculating the plot in (*d*) with the same color/marker scheme, and the total exposure is indicated along the bottom. Experimental data taken from EMPIAR 10061, beta-galactosidase. Scale bar 100 Å.

In Fig. 5 ▸(*e*) we show selected time points from a movie simulated using the continuum model (top row), experimental data (EMPIAR-10061; Bartesaghi *et al.*, 2014 ▸) in the middle row and the coarse-grained all-atom model calculated using the frozen plasmon method in the bottom row. As can be seen visually, the SNR_solvent_ is stronger for the continuum model, because the potential only has a DC component. This dependence on the PSD is also emphasized when comparing Figs. 5 ▸(*a*) and 5(*b*) that have the same average intensity in the solvent region, but very different SNR_solvent_ values. To quantify this effect, we calculated SNR_solvent_ as in Equation (2), defining the solvent region by the white portion of the mask in Fig. 5 ▸(*c*) and the protein as the central black region. The results are plotted in Fig. 5 ▸(*d*) where the final SNR_solvent_ is about a factor of two too strong using the continuum model, while our model closely matches that of experimental data.

### 3.6. Modeling the solvent envelope   

Having shown that we can simulate images with realistic SNR_solvent_ by using the integrated form of the scattering potential in conjunction with the frozen plasmon method, we now turn our attention from accurately simulating the solvent and inelastic losses to examining more nuanced components of the forward model by comparison with experimental images using the matched filter, with the output (SNR_mf_) defined in Equation (3).

**Figure 6:**
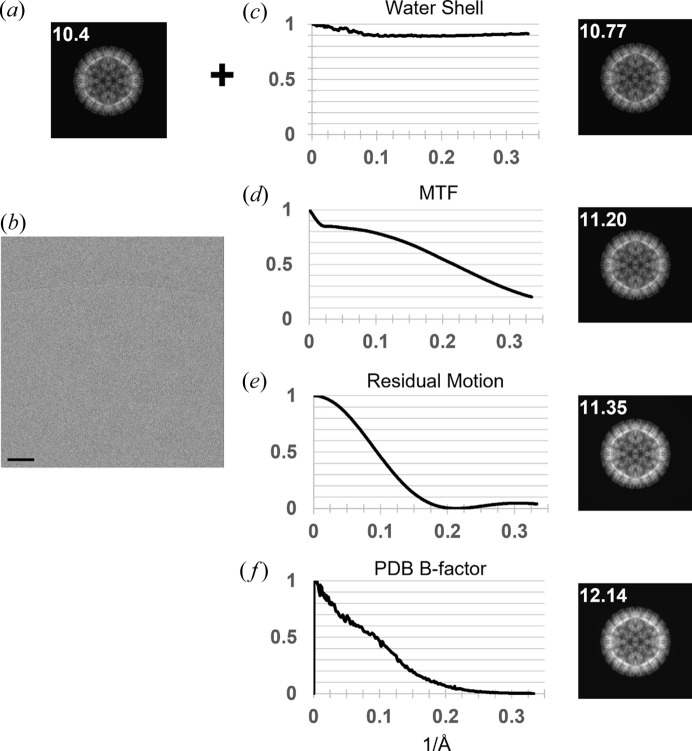
(*a*) Projection of simulated rotavirus DLP, using PDB entry 3gzu, with the average peak SNR_mf_ from 180 DLPs overlaid in white. (*b*) Example image from movie frame two, with only 1.5 e^−^ Å^−2^ cumulative exposure. (*c*) Ratio of the rotationally averaged power spectrum of a simulation with solvent embedding to the base simulation as in (*a*). (*d*) Ratio of the rotationally averaged power spectrum of a simulation with solvent embedding and detector MTF to the previous simulation as in (*c*). (*e*) Ratio of the rotationally averaged power spectrum of a simulation with solvent embedding, detector MTF and residual intra-frame motion blur to the previous simulation as in (*d*). (*f*) Ratio of the rotationally averaged power spectrum of a simulation with solvent embedding, detector MTF, residual intra-frame motion blur and PDB *B*-factors to the previous simulation as in (*e*). Scale bar 500 Å.

As a baseline, we calculate a ‘perfect’ model *in vacuo*, with the projected scattering potential of the rotavirus double-layer particle (DLP, PDB entry 3gzu; Chen *et al.*, 2009 ▸) shown in Fig. 6 ▸(*a*). We used this model to search a single early frame from 18 DLP movies, each with cumulative exposure of 1.5 e^−^ Å^−2^, *e.g.* Fig. 6 ▸(*b*). At this low exposure, we assume there to be no significant radiation damage. The average SNR_mf_ from 180 DLPs in these 18 movie frames is 10.4.

Biological macromolecules do not exist in a vacuum; they reside in a low-resolution ‘hole’ in the solvent, which impacts subsequent analysis as discussed in detail by Shang & Sigworth (2012 ▸). We incorporate their hydration radius model into the simulator by tracking the smallest distance to any non-solvent molecule and weighting any nearby solvent with a probability distribution defined by normalizing Equation (1) from their paper. We note that the parameter ‘r3’ in Table 1 of Shang and Sigworth should be ∼3.0, not 1.7. Additionally, we use the average of the coefficients they report for polar and non-polar residues, as the simulator currently only implements isotropic potentials for neutral atoms.

When simulating isolated macromolecules to use for comparison with experimental images, we weight the average water potential by this probability distribution, with an exponential decay starting at 4 Å into the bulk solvent. This exponential decay is added because our knowledge of the sample rapidly decays to zero beyond the particle of interest. This produces an effect similar to the *ad hoc* model suggested previously by Henderson & Mcmullan (2013 ▸). When simulating images, the probability distribution is applied to individual pseudo-water molecules as described next.

To illustrate the effect of applying the solvent envelope discussed in previous sections, we plot the rotationally averaged PSD of 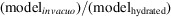 in Fig. 6 ▸(*c*). We observe that the spectral damping has little effect at low resolution (<20 Å) and is relatively constant at higher resolution (>10 Å). To quantify the effect, we include the SNR_mf_ overlaid with the projected density in Fig. 6 ▸(*c*).

### 3.7. Modeling other imaging effects that shape the signal distribution   

Subsequent panels in Fig. 6 ▸ show the rotationally averaged PSD of 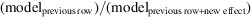 . The detector MTF reproduces the model applied, which we derived from the work by Ruskin *et al.* (2013 ▸). We chose this parameterization of the detector MTF because it also incorporates the coincidence-loss due to electron counting errors via depression of the DC component of the Fourier transform of 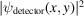 .

To account for blurring due to residual intra-frame specimen motion, we applied a Fourier space damping envelope, which for uniform motion is trivially a sin*c* function:

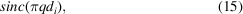

where *d_i_* (Å) is the real space displacement vector and *q*(Å^−1^) is the spatial frequency vector [for experimental observation of this *sinc* function, we refer the reader to the work by Frank (1969 ▸)]. Less trivial is determining the intra-frame specimen motion for which we only know a lower bound, estimated as the average of the displacements between the two neighboring frames. In Fig. 6 ▸(*e*) the modulation along the direction of motion is plotted (this would drop off to no modulation in the perpendicular direction). Lastly, we include the per-atom *B*-factors from the PDB file and plot the effective envelope in Fig. 6 ▸(*f*). Rather than trying to link these *B*-factors to any specific physical phenomena, we suggest they simply account for uncertainty in the modeled atomic coordinates that arise from a variety of factors.

By accounting for the solvent envelope, detector MTF, residual intra-frame motion and atomic model building uncertainty, we were able to increase the average SNR_mf_ from 10.4 to 12.14. Since the SNR_mf_ is expected to increase with the square root of the particle mass (Rickgauer *et al.*, 2017 ▸), this effectively reduces the mass limit of detection by ∼1.3×. We found that applying additional positive or negative *B*-factors only made the average score worse, indicating the model is reasonably accurate.

### 3.8. Assessing the radiation damage model   

Having developed a model that maximizes agreement with images lacking substantial radiation damage, we focus on radiation damage, long known to be *the* limiting factor in cryo-EM (Hayward & Glaeser, 1979 ▸). By studying movies recorded in the TEM, an analytical function modeling the effects of radiation damage on the coherent SNR_image reconstruction_ as a Fourier space filter – ξ(*q*) – was described by Grant & Grigorieff (2015 ▸). Given that radiation damage is specimen-dependent, the analytical model of Grant and Grigorieff will only strictly apply to the rotavirus VP6 capsid protein, and not to nucleic acids, for example. Alternatively, radiation damage may be modeled along with other blurring factors, for example, uncorrected motion blur, using exposure-dependent *B*-factors (Bartesaghi *et al.*, 2018 ▸; Scheres, 2014 ▸).

To quantify the accuracy in modeling radiation damage using the analytical model, ξ(*q*), we combine it with Equation (15) since the blurring due to residual intra-frame motion will be worse initially when the highest-resolution information is still present:

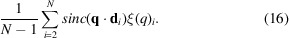

**Figure 7:**
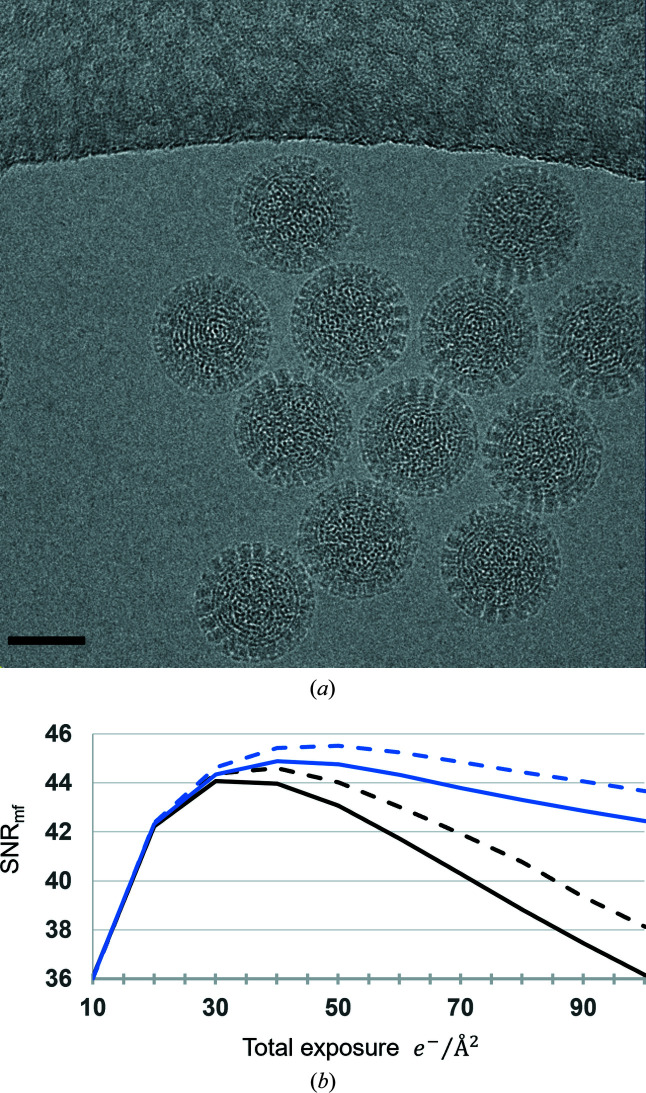
(*a*) Example image with a total exposure of ∼91 e^−^ Å^−2^. Rotavirus DLPs from the data set used in this analysis, kindly shared by Dr Tim Grant. (*b*) Average SNR values as a function of total exposure. Solid black line: simple averaging of movie frames, no exposure filtering. Dashed black line: image summed from exposure weighted movie frames. Solid blue line: reference simulated with cumulative exposure filter as would normally be applied to a movie. Dashed blue line: both image and reference exposure filtered. Red square: maximum SNR attainable using a single *B*-factor to represent all envelopes. Scale bar 500 Å.

Here we model what is observed in the images as the average over *N* movie frames 2 − *N* such that the accumulated exposure ranged from 10 to 100 e^−^ Å^−2^. A representative image is shown in Fig. 7 ▸(*a*). We then measured the average SNR_mf_ of the DLPs plotted in Fig. 7 ▸(*b*) with no exposure filtering (solid black line), exposure filtering applied to the image (dashed black line), exposure filtering applied to the simulated molecule (solid blue line) and exposure filtering applied to both data and model (dashed blue line). We found the largest increase in SNR_mf_ using a total exposure of 50 e^−^ Å^−2^ when applying the exposure filter to both the image and during simulation of the reference.

## 4. Discussion   

Our simulator implements the most thorough forward model for calculating the interaction between high-energy electrons and radiation-sensitive biological samples demonstrated to date. The improvements described here result from an approximate description of the changes in the specimen due to deposition of energy via inelastic scattering during imaging, incorporating a model for the solvent and its motion, as well as radiation damage. This added accuracy in simulating the molecular density produces more realistic image simulations for algorithmic development, but just as importantly, it provides a means to investigate the behavior of complex biological specimens in atomic detail using matched filtering via 2D template matching.

Since the output of the matched filter is sensitive to the PSD of the signal, we can quantify the accuracy of our image formation/damage model by measuring the change in SNR_mf_. We found that modeling the water envelope, detector MTF, residual intra-frame motion blur and atomic modeling uncertainty resulted in a higher SNR_mf_ than could be obtained by optimizing a single *B*-factor. This analysis is limited by the fact that we cannot strictly disentangle changes to the signal from different envelopes that could be mutually compensatory, though this may not be too severe a problem given the differences in the envelopes shown in Fig. 6 ▸(*a*). More careful consideration of the impact of different spatial frequencies on SNR_mf_ may prove useful in addressing this limitation in future work. In particular, we know high-spatial frequency signal strongly affects the SNR_mf_, but it is unclear how blurring effects not considered in detail here, *e.g.* axial coma, coherence of the electron beam or other higher-order aberrations may impact the contribution of high-spatial frequency to the SNR_mf_

We show that modeling the time dependency motion of the solvent is required to produce contrast that agrees well with experimental images. Our explicit solvent model, while coarse-grained, allows us to accurately reproduce attenuation due to inelastic losses and spatially variable amplitude contrast, and based only on the atomic species and local mass thickness in the simulated specimen. In principle, any configuration of atoms can be simulated by supplying an appropriate atomic coordinate file to the simulator. In practice, variable solvent thickness, protein fragments and other sources of structural noise, like regions of hexagonal ice could be included directly in the simulator, however, we leave this for future work.

A core idea in this work is to include recent empirical observations and measurements into the forward model. It may be possible to further improve the modeling of the molecular envelope by considering small-angle X-ray scattering data to complement the molecular dynamics information used here. It may also be important in the future to extend our model to include polar or charged atoms, which would change the character of the molecular solvent envelope. Modeling charged atoms would also enable us to include salts, which we expect to scatter strongly given their relatively high atomic numbers, as well as altering the local structure of the water molecules. This local ordering, however, would add yet another layer of computational complexity as it would require an anisotropic scattering factor and motion model. We note this directional modeling would also be required to model lipids accurately.

Another aspect of modeling charged atoms that may prove useful in the future is the ability to model atom- or residue-specific radiation damage. For example, acidic amino acid side chains seem to disappear rapidly in cryo-EM reconstructions made with increasing exposure (Barad *et al.*, 2015 ▸; Bartesaghi *et al.*, 2014 ▸). The disappearance of negatively charged chemical groups is likely to also be partially related to contrast inversion observed with negatively charged atoms (Yonekura *et al.*, 2018 ▸). Modeling charged atoms may require considering the immediate chemical environment of a residue, which would present considerable computational and theoretical challenges. To predict how much we expect the SNR_mf_ to improve with added exposure will require a more complete understanding of how object features with a given spatial frequency contribute to SNR_mf_ as well as how those features degrade over time.

Modeling the solvent presents computational challenges due to the sheer number of atoms we need to represent, which required a simplification to treat all solvent molecules as identical pseudo-waters. Even with these simplifications, we show a considerable improvement in matching SNR_solvent_ to experimental data and expect this to improve the ability of models (artificial neural networks especially) trained on simulated data to generalize more readily to experimental data.

In addition to modeling the solvent molecules as identical pseudo-waters, we addressed this computational cost via multi-threading in C++. Even so, the bulk of the calculation is spent on calculating the solvent and the Fourier transforms used during wavefunction propagation. To simulate more complex solvent models, or tilted samples, which will have a substantially larger number of slices to propagate, a GPU implementation may be beneficial for future work.

## 5. Conclusions   

Here we have presented an accurate forward model describing sources of signal attenuation and show how modeling the spectral characteristics of that attenuation improves the output of the matched filter (SNR_mf_ as used in template matching for the detection of molecules in cryo-EM images). The SNR_mf_ is in turn directly related to the mass limit for detection; any improvement in our forward model results in being able to detect smaller particles, which will expand the capacity of template matching in visual proteomics. The increased SNR_mf_ due to modeling radiation damage is encouraging but should likely be modeled more accurately for template matching. We also suggest that our model for inelastic scattering could be improved by direct comparison to experiment using the matched filter, by incorporating atom-type specific loss in template generation.

Taken together, these results suggest that other modifications to the template that result in a better match to the experimental data can further improve the SNR_mf_; for example, some amino acid side chains are affected more strongly by radiation damage than others, *e.g.* aspartate and the di­sulfide bond of cystine. These details could be incorporated into a new atom-specific damage model in future work.
